# Community-based intervention for cervical cancer screening uptake in a semi-urban area of Pokhara Metropolitan, Nepal (COBIN-C): study protocol for a cluster-randomized controlled trial

**DOI:** 10.1186/s13063-021-05049-3

**Published:** 2021-01-26

**Authors:** Aamod Dhoj Shrestha, Dinesh Neupane, Sarita Ghimire, Christine Campbell, Per Kallestrup

**Affiliations:** 1 Center for Global Health, Department of Public Health, Aarhus University, Aarhus, Denmark; 2 COBIN, Nepal Development Society, Bharatpur, Nepal; 3 Johns Hopkins Bloomberg School of Public Health, Baltimore, MD USA; 4 Nepal Cancer Care Foundation, Lalitpur, Nepal; 5 Usher Institute, University of Edinburgh, Edinburgh, EH8 9AG UK

**Keywords:** Female community health volunteers, Community health worker, Non-communicable diseases, Cervical cancer screening, Community-based, Randomized controlled trial, Nepal

## Abstract

**Background:**

Previous studies suggest that health intervention designed to increase cervical cancer screening has been effective to reduce cervical cancer incidence and mortality. The aim of this study is to determine the effect of a home-based health education intervention for increasing cervical cancer screening uptake delivered by trained female community health volunteers (FCHVs), a category of community health worker in Nepal.

**Methods:**

A community-based, open-label, two-armed, cluster-randomized trial [seven clusters (geographical wards) randomized for the intervention, and seven for the control arm]. The participants are recruited from a population-based survey with a sample size of 884. Based on population proportion size, 277 women will be recruited for the intervention group and 413 women recruited for the control group. A 12-month community-based health education intervention will be administered mobilizing the FCHVs, based on the Health Belief Model. The primary outcome measure of the study will be the difference in percentage of cervical cancer screening uptake between the two study arms. The primary outcomes will be modeled by using mixed-effect logistic regression analysis.

**Discussion:**

COBIN-C is the first study investigating the effect of a community-based health education intervention by FCHVs on increasing cervical cancer screening uptake among women in Nepal. The purpose of this study is to develop and implement a home-based, culturally sensitive program to increase cervical cancer screening coverage at the community level.

**Trial registration:**

ClinicalTrials.gov NCT03808064. Registered on January 14, 2019.

**Supplementary Information:**

The online version contains supplementary material available at 10.1186/s13063-021-05049-3.

## Background

Cervical cancer is a common cancer among women in most low- and middle-income countries (LMICs) in Asia, Africa, and Latin America [1]. It is the major cause of cancer deaths among women in Nepal with an estimated 2942 new cases and 1928 deaths in 2018 [1]. Screening is one of the most effective tools for early diagnosis, prevention, and treatment [2, 3]. However, there are very limited tools and skilled health professionals available for cervical cancer screening in many LMICs, including Nepal. The World Health Organization (WHO) and the Alliance for Cervical Cancer Prevention (ACCP) recommend that countries, areas, or institutions seeking to initiate or strengthen cervical cancer screening programs should consider introducing or expanding visual inspection with acetic acid (VIA) until more appropriate and affordable HPV-based tests become available [4, 5]. A single-visit approach with VIA and cryotherapy has been shown to be a safe, acceptable, feasible and is a potentially efficient method of cervical cancer prevention in Nepal [6]. National guidelines for the prevention and screening of cervical cancer includes VIA as a screening method for cervical cancer [7]. Studies reveal that participation of Nepalese women for cervical cancer screening is still low [2.8% (25–64 years); 1.5% (15–49 years); 5.4% (30–65 years)] [8–10]. Thus, there is a need for appropriate, cost-effective, and sustainable interventions to increase VIA screening uptake at the primary health care level across Nepal.

Studies suggest that mobilizing lay health workers and empowering them with integrated training could contribute in expansion of the knowledge base on non-communicable diseases (NCDs) at the community level and harmonize the community-based interventions [11–13]. In Nepal, female community health volunteers (FCHVs) have carried out community-based public health initiatives for more than 25 years and are willing to contribute to help mitigate NCDs [14]. FCHVs, a category of community health workers in Nepal, are married women, locally selected and trained through the Ministry of Health and Population. Previous studies demonstrated that FCHVs are able to screen for diabetes and hypertension: this has provided a favorable platform to explore the possible roles of FCHVs for increasing cervical cancer screening uptake [15, 16].

Mobilizing FCHVs to educate women and encourage them to participate in cervical cancer screening, and thus strengthening grass root level primary health provision, could be an acceptable and culturally appropriate intervention, which needs to be tested. Furthermore, it may contribute in the implementation of the National Cervical Cancer Screening and Prevention policy and ultimately help to reduce cervical cancer mortality in Nepal. The health promotion package will be a simple, feasible and effective approach.

In our study, we will conduct a community based cluster-randomized controlled trial (cRCT) to explore the roles of FCHVs in educating and empowering women through home visits to increase the uptake of VIA screening. Therefore, the primary outcome of the study is to increase the cervical cancer-screening uptake in the study area.

## Methods

This manuscript adheres to the Consolidated Standards of Reporting Trials (CONSORT) 2010 statement: extension to cRCT [17].

### Study design and setting

The proposed trial is a community-based, 12-month, open label, two-arm cRCT with an equal allocation of clusters (geographical wards) between intervention and control arms in a semi-urban area of Pokhara Metropolitan city (former Lekhnath municipality, administratively divided in to 15 wards). It is one of the two areas where the Cervical Cancer Screening and Prevention (CCSP) pilot program was first initiated in Nepal. The total population of Lekhnath municipality is 59,498 (female 32,104 and male 27,394) with the total number of households being 14,958 as per the 2011 census [18]. Each ward will be considered as one cluster. Although the study area is composed of 15 clusters, only 14 will be selected for the cluster randomization (one cluster that is comparatively different from others in socio-demography and health service availability is excluded). Allocation of the clusters into intervention and control arms is described below. The study area is limited to health services comprising one recently upgraded 25-bedded hospital from a primary health care center, three health posts, and six urban health care centers. According to the District Public Health Office, Kaski in 2018, there were 123 FCHVs in the municipality.

### Participants

**Fig. 1:**
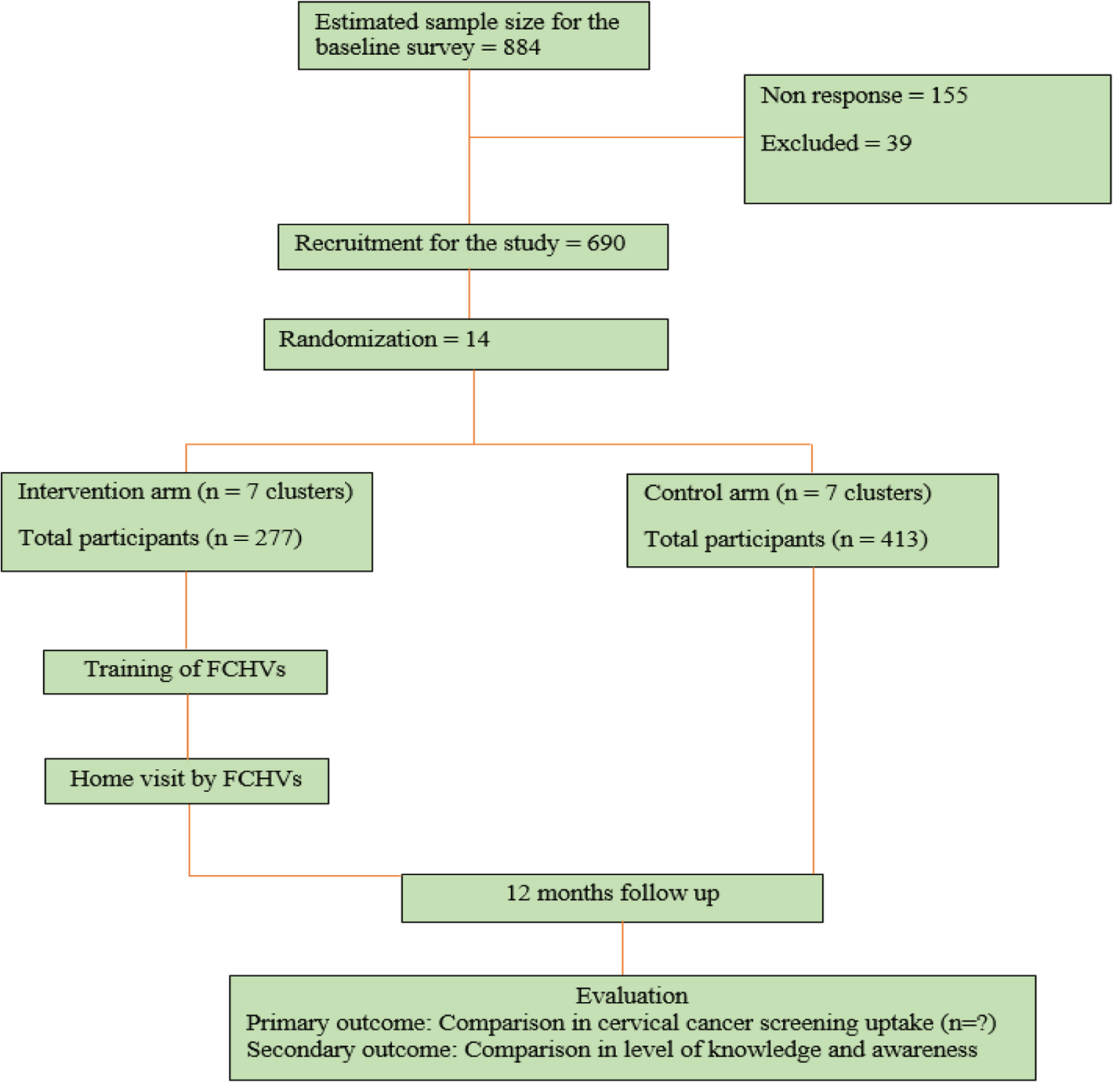
Planned flow chart of participants

The populations targeted in our study area are women aged 30–60 years (the age range recommended for VIA screening in Nepal), listed in the Community-Based Intervention for Non-communicable diseases study for hypertension (COBIN-H) [7, 16]. We will invite all the selected women participants for a baseline survey. During the baseline survey, knowledge, attitude, and screening practice on cervical cancer will be obtained and eligible women will be invited to participate in the trial. The flow of trial participants is shown in Fig. 1, which includes the number of participants eligible, surveyed, excluded, recruited, randomized, and analyzed for the primary outcome. The SPIRIT checklist for this trial is provided as an Additional file 1.

### Recruitment

A sampling framework was adopted from the COBIN-H trial that has taken a population framework of all eligible participants from the voter list prepared by the Election Commission in 2017 [16]. As of 31 December 2018, all the women of age group 30–60 years were included in the study. Trained women public health professionals (data enumerators) will carry out a baseline survey. The data enumerators will receive 4 days of intensive training before data collection. The survey tool is adapted from a joint survey report of Family Health Division of Nepal’s Ministry of Health and United Nations Population Fund (UNFPA) [8, 19–21]. It includes questions on survey information, consent, socio-demographic information, questions related to sexual and reproductive health, health-seeking behavior, knowledge, attitude and cervical cancer screening practice, and sign and symptoms. The questionnaire was modified as per the Nepalese settings, translated to the Nepali language and back to English to ensure content validity. Baseline data will be used for possible confounder adjustment and stratification by effect modifiers. The data enumerators will identify eligible participants, ask about their willingness to participate in the cRCT, receive informed consent, and recruit in the trial. The required number of participants will be randomly selected from those willing to participate in the cRCT: FCHVs will visit each household, meet the selected participant, and obtain consent to participate in the trial.

### Randomization

Clusters will be randomly assigned to intervention and control areas using a 1:1 allocation ratio. Randomization will be done before the participants are recruited. Research teams responsible for identifying potential participants, obtaining consent, and recruiting trial participants will be blinded to the participants’ allocation status. The following steps will be taken to avoid selection bias during random allocation: (a) clusters will be randomized only after the baseline survey, (b) clusters will not be withdrawn from or added to the study, and (c) a statistician from abroad who is not familiar to the study will randomize the clusters.

### Inclusion and exclusion criteria

Women aged 30–60 years who are listed in COBIN-H and who indicated they planned to reside in a cluster in the study area for at least the next 12 months are eligible for inclusion in the baseline survey and for the invitation to participate in the study. Women who are severely ill, pregnant, less than 6 weeks after delivery, who are already diagnosed with cervical pre-cancer and cancer, with a history of total hysterectomy, as well as those who decline to participate in the study or unable to complete the interview, will be excluded from the study.

### Outcomes

#### Primary and secondary outcomes

The primary outcome of the study is percentage change in cervical cancer screening uptake from baseline to follow-up in the intervention group compared to the control group in a 12-month home-based health education intervention. The baseline survey will be conducted before the cluster randomization, and a follow-up survey of similar method will be conducted after 12-month intervention. The response of each participant for cervical cancer screening in the baseline survey and the follow-up survey will be compared to evaluate the percentage. In addition, FCHV will provide a referral slip to the participants in the intervention group (keeping a copy of the referral slip to submit to the field supervisor) during the home visit, which they take with them and submit to the health facility for VIA screening. The referral slip contains study code, name, age, address, and signature of both FCHV and the participant. FCHV will inform the participants about the health facility where the services are freely available. The key health personnel providing the VIA screening services are oriented about the study. Furthermore, confidentiality about the participants’ information will be maintained throughout the intervention. The field supervisor will collect the referral slip to confirm that the participants have received VIA screening and results from all the health facilities of the intervention ward as a reference to the participants’ response.

The secondary outcomes of the study are change in the level of knowledge and awareness among women on cervical cancer screening and prevention. The response of each participant for knowledge and awareness on cervical cancer screening in the baseline survey and the follow-up survey will be compared to evaluate the change using the validated questionnaire [19, 20].

### Sample size calculation

The sample size for the baseline survey is calculated based on the method suggested by the WHO STEPWISE Approach [22]: for our study, we calculate 884 participants with 20% non-response. A systematic random sampling method will be performed as a sampling technique. For the power calculation, we took reference from a quasi-experimental study from Nigeria that reported an effect size of 5% between intervention and control group during a 13-week period [23]. We expect an effect size of 10% increase in our study with a 1-year follow-up where FCHVs are mobilized to conduct home visit counseling once every 3 months and the intervention takes place in an ongoing community-based program for non-communicable diseases from 2014 [16]. Assuming, 20% loss to follow-up and ICC of 0.01 and seven clusters per arm will provide 90% power resulting to 49 women per cluster. The total sample size needed for the intervention study is 690 that will be recruited from the 884 participants (baseline survey), which is enough to obtain 90% power.

### Interventions

After randomization, the FCHVs in intervention wards will receive training coordinated by the research team in collaboration and close supervision of the local health authority. The training manual is designed under the guidance of experts in cervical cancer and public health from the national and international organizations. The training manual is based on the following: training of community health workers, WHO, 2017 training manual [24] and Cervical Cancer Screening and Prevention (CCSP) A Reference Manual 2015 [25]. The FCHVs will receive a 3-day training package highlighting: (a) an introduction to NCDs and cervical cancer, (b) cervical cancer causes and risk factors, (c) cervical cancer screening and service availability, (d) providing health education and counseling women (30–60 years), and (e) recording, reporting, and follow-up. An overview of program content is presented in Table 1. Educational sessions are guided by the use of the Health Belief Model [26], and training materials will be reviewed and validated by experts and major stakeholders. The developed training materials will be pretested with the FCHVs from a nearby area not included in the study. In addition, the FCHVs will also receive health education materials (pamphlets), referral cards, and a recording register during the training session.

**Table 1:** Session plan for FCHV training

Day	Session	Main contents
**Day 1**		
	Introduction to non-communicable diseases (NCD)	What is NCD and its situation in Nepal? What are the different types of NCDs? What are the risk factors for NCDs? How can we prevent NCDs?
	Cervical cancer Causes and symptoms	Have you ever heard of cancer? What is cancer? What is cervical cancer? What causes cervical cancer? What are the symptoms of cervical cancer? How does the community perceive cervical cancer?
	Discussion	Questions/answers and doubt clearance
	Evaluation	First day evaluation and FCHV selection
**Day 2**		
	Cervical cancer Causes and symptoms	What causes cervical cancer? What are the symptoms of cervical cancer? Human papilloma virus and it’s transmission Who are at risk of having cervical cancer?
	Cervical cancer screening and prevention	What to do to prevent cervical cancer? What are the different types of screening methods? Visual inspection with acetic acid (VIA) What is the recommended age group for VIA screening? Who does the VIA screening? Service delivery points and cost Role of FCHV to prevent cervical cancer Myths about VIA screening.
	FCHV home visit	Planning for home visit Number of visits and timing Rapport building Reason for the visit and the health education session Recording, reporting
	Interactive learning	Flip chart topics FCHV posture, body language, preparedness, proactive towards the situation, voice Detailing the flip chart
**Day 3**		
	Recording register	Explain the content of the recording register How to prepare and fill the recording register? Name, age, location of the participants and FCHV Submission of completed register to the research officer
	Pamphlet	Explain the content of the pamphlet How to stress the points in the pamphlet What to suggest to the participants who are not able to read the pamphlet?
	Referral card	Explain the content of the referral card How to fill the referral card? Name, age, location, and health facility address for referral Communication with the health facility
	Scenario planning	Role play
	Household selection and list preparation	Selection of the household for home visit according to FCHVs working area.

At the end of the training, FCHVs will assign the number of households that have to be visited based on the baseline survey. Participants in the intervention clusters will receive a 12-month, home-based, health education package administered by FCHVs in a household setting. FCHVs will visit each household, meet the selected participant, receive approval, and provide the health education. On average, one FCHV will meet 18 participants three times a year. FCHV will complete the three visits to provide health education even if the participants have already received VIA screening. During the visit, FCHVs will deliver the health education intervention, to encourage and empower women for cervical cancer screening uptake available free of cost at their local health facility. They will also conduct two reinforcement visits every 4 months from the first visit (three home visits in a year).

Health workers conducting cervical cancer screening at the local health facility in the intervention group will receive orientation about the study to make sure that the women attending the health facility receive screening. They will ensure that the equipment is functional and reagents are available, record information, provide psychosocial counseling to the case positives, and refer for further confirmation and treatment. The recorded information at the health facility will be tallied using the referral slip to confirm women’s participation for cervical cancer screening. The collected data will be kept strictly confidential and will only be accessed by the members of the trial team.

Each participant is assigned an individual trial identification number, which is securely stored. The principal investigator reserves the right to the data set and could share upon reasonable request.

There will be no special criteria for discontinuing or modifying allocated interventions. Furthermore, the implementing community-based intervention for cervical cancer screening uptake will not require alteration to usual care pathways (including use of any medication) and will continue for both the trial arms.

A field supervisor will routinely visit each FCHV in order to maintain motivation and collection of records from the health facilities. The supervisor also provides ad hoc help to the FCHVs when needed during the intervention period. We will also use a supervision checklist to track and update the knowledge and skill levels of FCHVs as well as to ensure fidelity to the study protocol.

In the control clusters, participants will receive “usual care.” The usual care means the current health education and promotion practices for cervical cancer screening at the community level provided by the government health system. They will not receive further contact, information, or educational materials from FCHVs until the 12-month assessment.

### Minimization of contamination

The risk and level of contamination will be monitored at the levels of FCHVs and participants. FCHVs will be instructed not to share information about the study and not to provide any support to people from other clusters in the community, other than the ones who are assigned. The risk of contamination will also be minimized by the cluster design, whereby the intervention and control clusters will be geographically separated and the chance of intervention cluster participants regularly meeting control cluster participants will be negligible. All possible means of contamination between intervention and control participants will be collected during the follow-up survey and adjustments will be made for estimating the effect.

### Intervention fidelity

The intervention is designed under the guidance of experts in cervical cancer and public health from the national and international organizations. The subject experts and major stakeholders reviewed and validated the training materials. The education session were delivered under the close supervision of the local level health authority and the research team, who will monitor the intervention. The Data Safety and Monitoring Board (DSMB) along with a member from the Nepal Health Research Council will evaluate the progress of the trial, including periodic assessments of data quality and timeliness, participant recruitment, accrual and retention, participant risk versus benefit, performance of the trial site, and other factors that can affect study outcome. Each FCHV in the intervention cluster will be advised of the importance of following the intervention steps and content during the training session. The field supervisor will monitor FCHVs tasks. FCHVs will deliver the services guided by a register that keeps them reminded of stages of the intervention and will report every 4 months.

### Data analysis

STATA version 15 software (StataCorp, College Station, TX, USA) will be used for quantitative analysis following the Consolidated Standards of Reporting Trials (CONSORT) guidelines for cluster-randomized trials. Flowcharts (Fig. 1) will include the number of participants seen at each stage of the trial, eligible, randomized, and analyzed for the primary outcome. In the initial analysis, we will compare baseline characteristics of enrolled participants in the study arms and follow-up status. We will use mixed-effect logistic regression to compare between the groups as the outcome (the primary outcome would be the difference in the proportion of women who participate in the cervical cancer-screening program between intervention and control within 1 year). Comparison of the knowledge scores between baseline and follow-up, after 1-year intervention, will measure the knowledge level. Estimation will take into account the effect of potential confounders (age, ethnicity, education, parity, income, location, etc.).

## Interim analyses and stopping rules

There is no plan for interim analysis, as we do not expect a situation that would lead us to stop the study. However, in the event of an extreme situation such as a natural disaster, a conflict, or duplication of similar interventions in our control area, we will temporarily stop the study to assess the implications on the study design.

## Trial management

We have a robust mechanism to ensure data quality. At first, the data enumerators will receive intensive training on the process of data collection. The principal investigator will monitor the data enumerators on a day-to-day basis. The research assistant assigned for data entry will inspect the data manually and enter the data in EpiData software file (EpiData Association, Odense, Denmark). Double entry for 10% of the data will qualify potential errors in data entry quality. The research officer will crosscheck the data quality on the spot and in our field office for any incomplete, inconsistent, and invalid data. Any deviation in the data quality will require recollecting in the field.

## Indemnities

The study carries minimal risk to the study participants, which is why we have no compensation or insurance plan for the participants. However, we will provide medical insurance and US$5 per home visit to FCHVs for transportation expenses.

## Publication plan

The results of the trial will be published in a peer-reviewed journal.

## Discussion

The COBIN-C study is the first investigation of a community-based intervention to increase cervical cancer screening uptake in Nepal. We expect that a community-based, culturally tailored education intervention delivered by community health workers such as FCHVs will play a key role in increasing cervical cancer screening uptake in the study area. Indeed, community health workers can play an important role in understanding the cultural barriers including community health practices, cultural identity, and coping practices that can promote positive health outcomes [27].

Furthermore, studies from Iran, Kenya, and Nigeria have reported health education interventions and behavior change frameworks provide an effective base for cervical cancer prevention [23, 28–30]. In our study, FCHV in the intervention group will make three home visits to educate and empower women for cervical cancer screening uptake that is available free of cost at the local health facilities. Previous study reveals that appropriate and structured training program motivates FCHVs to contribute in addressing non-communicable diseases [14].

The health promotion package is a simple, feasible, and potentially effective approach. It may contribute in the implementation of the National Cervical Cancer Screening and Prevention policy and help reduce cervical cancer mortality in the long term in Nepal and if successful may be adapted to similar settings elsewhere.

## Trial status

The trial is now closed to participant accrual, but the trial is ongoing. The endpoint assessment of all the participants will be completed by the end of August 2021. The schedule of enrollment, interventions, and assessments is presented in Table 2. The protocol has been submitted for publication after the end of recruitment due to manuscript preparation and author’s travel plan.

**Table 2:** Schedule of enrolment, interventions, and assessments

	Study period						
	Enrolment	Allocation	Post-allocation				Close-out
Timepoint (months)	***3***	1	***1***	***4***	***4***	***4***	***2***
**Enrolment:**							
**Eligibility screen**	**x**						
**Informed consent**	**x**						
***Informed consent with FCHVs***	**x**						
**Allocation**		**x**					
**Interventions:**							
***Training of FCHVs***			**x**				
***Home visit by FCHVs***				**x**	**x**	**x**	
**Assessments:**							
*Baseline variable* *Socio-demographic (age, education, ethnicity, monthly income, marital status), pregnancy, sexual, and reproductive characteristics*	**x**						**X**
***Outcome variable*** *# of women screened for cervical cancer*	**x**						**X**

The protocol version number and date—V1, 28 December 2020.

## Supplementary Information

**Additional file 1.** SPIRIT 2013 Checklist: COBIN-C.

**Additional file 2.** Questionnaire: COBIN-C.

## Data Availability

Data set and informed consent forms will be available upon reasonable request to the corresponding author ADS.

## Abbreviations

cRCT: Cluster randomized controlled trial
FHD: Family Health Division
FCHVs: Female community health volunteers
LMICs: Low- and middle-income countries
UNFPA: United Nations Population Fund
WHO: World Health Organization

## References

[1] International Agency for Research on Cancer, Cancer Today. Global cancer observatory, Lyon, France. 2018. https://gco.iarc.fr/today/fact-sheets-populations. Accessed 6 April 2020.

[2] Sankaranarayanan R, Nene BM, Shastri SS (2009). HPV screening for cervical cancer in rural India. N Engl J Med.

[3] Denny L, Kuhn L, De Souza M, Pollack AE, Dupree W, Wright TC (2005). Screen-and-treat approaches for cervical cancer prevention in low-resource settings: a randomized controlled trial. J Am Med Assoc.

[4] WHO guidelines for screening and treatment of precancerous lesions for cervical cancer prevention. World Health Organization. 2013. http://apps.who.int/iris/bitstream/10665/94830/1/9789241548694_eng.pdf. Accessed 15 April 2020. 24716265

[5] Cervical Cancer prevention Fact Sheet. Alliance for Cervical Cancer Prevention. 2004. https://screening.iarc.fr/doc/RH_fs_risk_factors.pdf. Accessed 15 April 2020.

[6] Gaffikin L, Blumenthal PD, Emerson M, Limpaphayom K. Royal Thai College of Obstetricians and Gynaecologists (RTCOG)/JHPIEGO Corporation Cervical Cancer Prevention Group [corrected]. Safety, acceptability, and feasibility of a single-visit approach to cervical-cancer prevention in rural Thailand: a demonstration project. Lancet. 2003;361(9360):814–20. 10.1016/s0140-6736(03)12707-9 12642047

[7] Family Health Division. National Guideline for Cervical Cancer Screening and Prevention in Nepal. Department of Health Services, Kathmandu: Government of Nepal; 2010.

[8] Selected Reproductive Health Morbidities among Women attending Reproductive Health Camps in Nepal. United Nations Population Fund and Family Health Division, 2016. https://nepal.unfpa.org/sites/default/files/pub-pdf/RH%20Morbidity%20study_0.pdf. Accessed 15 April 2020.

[9] Bruni L, Albero G, Serrano B, Mena M, Gómez D, Muñoz J, Bosch FX, de Sanjosé S. ICO/IARC Information Centre on HPV and Cancer (HPV Information Centre). Human papillomavirus and related diseases in Nepal. 2019. https://hpvcentre.net/statistics/reports/NPL.pdf. Accessed 15 April 2020.

[10] Ranjit A (2016). Awareness and prevalence of cervical cancer screening among women in Nepal. Int J Gynecol Obstet.

[11] Khatri RB, Mishra SR, Khanal V (2016). Female community health volunteers in community-based health programs of Nepal: future perspective front. Public Health.

[12] Kane S, Kok M, Ormel H, Otiso L, Sidat M, Namakhoma I, Nasir S, Gemechu D, Rashid S, Taegtmeyer M, Theobald S, Koning K (2016). Limits and opportunities to community health worker empowerment: a multi-country comparative study. Soc Sci Med.

[13] Winangnon S, Sriamporn S, Senarak W, Saranrittichai K, Vatanasapt P, Moore MA (2007). Use of lay health workers in a community-based chronic disease control program. Asian Pac J Cancer Prev.

[14] Neupane D, McLachlan CS, Gautam R (2015). Literacy and motivation for the prevention and control of hypertension among female community health volunteers: a qualitative study from Nepal. Glob Health Action.

[15] Gyawali B (2018). Community-based intervention for management of diabetes in Nepal (COBIN-D trial): study protocol for a cluster-randomized controlled trial. Trials..

[16] Neupane D (2016). Community-based intervention for blood pressure reduction in Nepal (COBIN trial): study protocol for a cluster-randomized controlled trial. Trials..

[17] Campbell MK, Piaggio G, Elbourne DR, Altman DG, for the CONSORT Group (2012). Consort 2010 statement: extension to cluster randomised trials. BMJ.

[18] National Population and Housing Census 2011(National Report). Central Bureau of Statistics. Kathmandu, Nepal. 2012. Page 42. https://cbs.gov.np/national-population-and-housing-census-2011national-report/. Accessed 14 April 2020.

[19] Thapa N, Maharjan M, Petrini MA, Shah R, Shah S, Maharjan N, Shrestha N, Cai H (2018). Knowledge, attitude, practice and barriers of cervical cancer screening among women living in mid-western rural, Nepal. J Gynecol Oncol.

[20] Touch S, Oh JK (2018). Knowledge, attitudes, and practices toward cervical cancer prevention among women in Kampong Speu Province, Cambodia. BMC Cancer.

[21] Improving data for decision-making: a toolkit for cervical cancer prevention and control programmes. World Health Organization, United States Centers for Disease Control and Prevention, CDC Foundation, George W. Bush Institute. 2019. https://www.who.int/ncds/surveillance/data-toolkit-for-cervical-cancer-prevention-control/en/. Accessed 23 April 2020.

[22] Noncommunicable diseases and their risk factors. STEPS Resources. World Health Organization, Geneva. 2014. http://www.who.int/chp/steps/resources/en/. Accessed 23 April 2020.

[23] Abiodun OA (2014). Impact of health education intervention on knowledge and perception of cervical cancer and cervical screening uptake among adult women in rural communities in Nigeria. BMC Public Health.

[24] Cervical cancer screening and management of cervical pre-cancers: training of community health workers. World Health Organization. Regional Office for South-East Asia. 2017. https://apps.who.int/iris/handle/10665/279798. Accessed 15 April 2020.

[25] Family Health Division. Cervical Cancer Screening and Prevention (CCSP) in Nepal. A reference manual. Department of Health Services, Kathmandu: Government of Nepal; 2015.

[26] Janz NK, Becker MH (1984). The Health Belief Model: a decade later. Health Educ Q.

[27] Glenton C, Scheel IB, Pradhan S, Lewin S, Hodgins S, Shrestha V (2010). The female community health volunteer programme in Nepal: decision makers’ perceptions of volunteerism, payment and other incentives. Soc Sci Med.

[28] Ghahremani L, Harami ZK, Kaveh MH, Keshavarzi S (2016). Investigation of the role of training health volunteers in promoting pap smear test use among Iranian women based on the protection motivation theory. Asian Pac J Cancer Prev.

[29] Gana GJ, Oche MO, Ango JT, Raji MO, Okafoagu NC (2016). Effect of an educational program on awareness of cervical cancer and uptake of Pap smear among market women in Niger State, North Central Nigeria. J Public Health Epidemiol.

[30] Rosser JI, Njoroge B, Huchko MJ (2015). Changing knowledge, attitudes, and behaviors regarding cervical cancer screening: the effects of an educational intervention in rural Kenya. Patient Educ Couns.

