# Supplementary material for: Community-based intervention for cervical cancer screening uptake in a semi-urban area of Pokhara Metropolitan, Nepal (COBIN-C): study protocol for a cluster-randomized controlled trial
Source: Trials. 2021 Jan 26;22:94. doi: 10.1186/s13063-021-05049-3 (PMC7836185; doi:10.1186/s13063-021-05049-3)
Supplement: Supplementary file 2 — Additional file 2. Questionnaire: COBIN-C. [file 13063_2021_5049_MOESM2_ESM.pdf]

COMMUNITY-BASED INTERVENTION FOR CERVICAL CANCER SCREENING UPTAKE IN A SEMI-URBAN AREA OF POKHARA METROPOLITAN, NEPAL: A CLUSTER RANDOMIZED CONTROLLED TRIAL (COBIN-C)

**Participants identification number** ☐☐☐☐☐☐☐☐			
**LOCATION AND CONSENT**			
**Location and date**		**Responses**	**Codes**
1.	House GPS	GPS coordinates: N _ _ º _ _ ’ _ _ ”<br>E _ _ º _ _ ’ _ _ ”<br>Altitude: _ _ _ _ _ meters<br>Date (DD/MM/YY): _ _ / _ _ / _ _	I1
2.	Ward number	……………	I2
3.	Interviewer ID	…..................	I3
4.	Date of completion of the interview	……………	I4

✂----✂------✂-----✂------✂-----✂------✂-----✂------✂-----✂------✂-----✂

	**Consent and name**	**Responses**	**Codes**
5.	Consent has been read and obtained	No 0<br>Yes 1 **If No stop here and write the reason for rejection**<br>**Reason:**	I5
6.	Time of beginning of interview in 24 hrs	Start ☐☐ : ☐☐<br>End ☐☐ : ☐☐<br>Hours Minutes	I6
7.	Family surname<br>(Both surname before and after marriage if applicable)		I7
8.	First name		I8
9.	Mobile and Landline phone number	Mobile …………. Phone …………	I9
10.	Do you have any plans to move for study/marriage/employment from your current residence in the coming one year?	No 0<br>Yes 1	I10
11.	Would you like to participate in this intervention study?	No 0<br>Yes 1	I11

Record and file identification information (I5 to I11) separately from the completed questionnaire.

	**Participants identification number** ☐☐☐☐☐☐☐☐		
**SOCIO DEMOGRAPHIC INFORMATION**			
**Questions**		**Responses**	**Codes**
12.	What is your Date of Birth?	☐☐■☐☐■☐☐☐☐ **B.S.**<br>☐☐■☐☐■☐☐☐☐ **A.D.**<br>**Day** **Month** **Years**	C1
13.	What is your age?	.... completed years	C2
14.	How many years did you spend for formal education?<br>(Don't count pre-primary education)	☐☐ completed years	C3
15.	What is the highest level of education you have completed?	Illiterate 0<br>No formal schooling 1<br>Less than primary school 2<br>Primary school completed 3<br>Secondary school completed 4<br>Higher secondary (10+2)/ PCL completed 5<br>Bachelor degree completed 6<br>Post graduate degree 7<br>Prefer not to answer 88	C4
16.	What is your ethnic background?	Dalit 1<br>Disadvantaged Janajatis 2<br>Disadvantaged non-dalit terai caste groups 3<br>Religious minorities 4<br>Relatively advantaged Janajatis 5<br>Upper caste groups 6<br>Prefer not to answer 88	C5
17.	What is your religion	Hindu 1<br>Buddhist 2<br>Christian 3<br>**If others specify in C6 other**	C6
		Others (specify) …………………………………	C6 other
18.	What is your current marital status?	Unmarried 1 **If single [1] skip to C9**<br>Married 2<br>Separated 3<br>Divorced 4<br>Widow 5<br>Live in together 6<br>Prefer not to answer 88	C7
19.	How old were you when you (first) got married?	Age ………………. (write the completed years)	C8
20.	Which of the following best describes your husband's main work status over the past 12 months?	Government employee 1<br>Non-government employee 2<br>Farmer/Agriculture 3	C9

		Daily wage worker 4<br>Foreign employment 5<br>Business 6<br>Others 7 **If others specify in C6 other**<br>Prefer not to answer 88	
		Others (specify) ……………………………………	C9 other
21.	If you are married, Is your husband currently living with you or abroad?	Unmarried 1<br>Husband at home 2<br>Husband abroad 3<br>Other 4	C10
22.	Which of the following best describes your main work status over the past 12 months?	Homemaker 1<br>Government employee 2<br>Non-government employee 3<br>Farmer/Agriculture 4<br>Daily wage worker 5<br>Business 6<br>Others 7 **If others specify in C11 other**<br>Prefer not to answer 88	C11
		Others (specify) ……………………………………	C11 other
23.	What is your approximate monthly income?	Daily ……………………<br>Weekly ……………………<br>Monthly ……………………<br>Prefer not to answer 88	C12
24.	What is your approximate family monthly income?	Daily ……………………<br>Weekly ……………………<br>Monthly ……………………<br>Prefer not to answer 88	C13

**PREGNANCY AND OTHER DISEASE RELATED QUESTIONNAIRE**			
**Questions**		**Responses**	**Codes**
25.	Do you have any family member who suffered from any type of cancer?	Yes ……………… type of cancer 1<br>No 0	P1
26.	Are you diagnosed with any chronic diseases?<br><br>(Multiple choice)<br><br>(Read the response)	Diabetes Yes 1 No 0<br>Hypertension Yes 1 No 0<br>COPD Yes 1 No 0<br>HIV/AIDS Yes 1 No 0<br>Cervical cancer Yes 1 No 0<br>Pre-cancer Yes 1 No 0<br>Hysterectomy Yes 1 No 0<br>**If 'Cervical cancer / pre-cancer / hysterectomy confirm again and note**	P2

27.	How many times have you been pregnant?	…………………. Times<br>(Including miscarriage, still birth & abortion)	P3
28.	Did you have any abortions, still births or miscarriages in the past?	Miscarriages ……..…………Times<br>Still births…………………. Times<br>Abortions ………………….Times	P4
29.	Are you pregnant now?	Yes …………………… 1 **If participant is pregnant note**<br>*write completed months*<br>No 0<br>Unmarried 55	P5
30.	How old were you at the time of your first pregnancy?	……………..……………Age<br>(Write the completed year)<br>Don't know 66<br>Don't remember 77<br>Unmarried 55	P6
31.	How many children do you have now?	Zero 1 **If the answer is zero or one child then skip to P9**<br>Total children ……………..<br>Son.…………<br>Daughter…………<br>Unmarried 55	P7
32.	What is the shortest birth interval between your children?<br>*(Consider the shortest birth interval if she has more than two children)*	☐☐<br>*(write the complete years)*<br>Unmarried 55	P8
33.	Do you know what is …………?	Condom Yes 1 No 0<br>Pills / Nilocon Yes 1 No 0<br>Sangini Yes 1 No 0<br>IUD Yes 1 No 0<br>Implant /Norplant Yes 1 No 0<br>Withdrawal Yes 1 No 0<br>Rhythm method Yes 1 No 0	P9
34.	Have you ever used …………….?	Condom Yes 1 No 0<br>Pills / Nilocon Yes 1 No 0<br>Sangini Yes 1 No 0<br>IUD Yes 1 No 0<br>Implant /Norplant Yes 1 No 0<br>Withdrawal Yes 1 No 0<br>Rhythm method Yes 1 No 0	P10
35.	Among the devices / method used, which one is the latest?	Condom 1<br>Pills / Nilocon 2<br>Sangini 3<br>IUD 4<br>Implant /Norplant 5<br>Withdrawal 6<br>Rhythm method 7	P11

		Other 8	
36.	How many weeks earlier, you have used that device / method?	……………………………. Weeks earlier	P12
37.	Do you smoke?	Yes 1<br>I was a previous smoker 2<br>No, I have never smoked 3	P13
38.	Do you use smokeless tobacco products? (E.g. Surti, Khaini, Gutkha)	Yes …………… (specify) 1<br>I used it earlier…………. (specify) 2<br>No I have never used 3	P14
39.	Do you drink alcohol?	Yes, regularly 1<br>Yes, occasionally 2<br>I used to drink earlier now stopped 3<br>Never 4	P15

HEALTH-SEEKING BEHAVIOR			
Questions		Responses	Codes
40.	What means of transportation do you use to the nearest health facility?	On foot 1<br>Bus 2<br>Motorbike 3<br>Others 4<br>Don't know 66 **If others specify in H1 other**	H1
		Others (specify) …………………………………….	H1 other
41.	How long time does it take you to reach the nearest health facility from your house?	………………………. (in minutes)	H2
42.	Where do you usually go first if you are sick, or to treat a general health problem?<br>(Check all that are mentioned)	Private clinic 1<br>Government clinic or hospital 2<br>Traditional healer 3<br>Clinic run by an NGO 4<br>Others 5 **If others specify in H3 others**	H3
		Others (specify) …………………………………….	H3 other
43.	How often do you generally seek health care at a clinic or hospital in the last five years? (Check one.)	Twice a year or more 1<br>Once per year 2<br>Less than once a year but at least twice in past 5 yrs 3<br>Once in past 5 years 4<br>Never in past 5 years 5<br>Others 6 **If others specify in H4 others**	H4
		Others (specify) …………………………………….	H4 other

KNOWLEDGE RELATED QUESTIONNAIRE ON CERVICAL CANCER			
**Questions**		**Responses**	**Codes**
44.	1. Have you ever heard about cervical cancer? Total score = 1	Yes 1<br>No 0 **If No [2] skip to KS1**	KC1
45.	2. If yes, how did you come to know about cervical cancer? (Multiple responses)<br><br>Total score =	Television 1<br>Radio 2<br>Newspaper 3<br>Health personnel 4<br>Relatives 5<br>Friends 6<br>FCHV 7<br>Others 8 **If others specify in KC2 others**	KC2
		Others (specify) ……………………………………	KC2 others
46.	3. What do you mean by cervical cancer?	Pain in cervix 1<br>Abnormal growth of the cells of cervix 2<br>Swelling in the cervix 3<br>4<br>Don't know 66	KC3
		Others (specify) ……………………………………	KC3 others
47.	4. Do you know the sign and symptoms of cervical cancer?	Yes 1<br>No 0 **If No [2] skip to KC6**	KC4
48.	5. What are the signs and symptoms of cervical cancer? (Multiple responses)	No symptoms at early stage 1<br>Blood spots or light bleeding between or following periods 2<br>Bleeding after sexual intercourse 3<br>Bleeding after menopause 4<br>Pain during sexual intercourse 5<br>Increased vaginal discharge 6<br>**If others specify in KC5 others**	KC5
		Others (specify) ……………………………………	KC5 others

49.	6. In your opinion, what are the risk factors for cervical cancer? (Multiple responses)	Human papillomavirus (HPV) 1<br>Smoking 2<br>Early marriage 3<br>Having many children 4<br>Having many sexual partners 5<br>Alcoholic drinks 6<br>Long-term oral contraceptive use 7<br>Low socio-economic status 8 **If others specify in KC5 others**	KC6
		Others (specify) …………………………………….	KC6 others
50.	7. Have you heard about Human Papilloma Virus infection?	Yes 1<br>No 2 **If No [2] skip to KC9**	KC7
51.	8. If Yes, tell me how is Human Papilloma Virus transmitted?	Air pollution 1<br>Water pollution 2<br>Sexual contact 3<br>Others 4 **If others specify in KC8 others**	KC8
		Others (specify) …………………………………….	KC8 others
52.	9. How can cervical cancer be prevented? (Multiple responses)	Avoiding multiple sexual partners 1<br>Avoiding multiple pregnancies 2<br>Avoiding early marriage 3<br>Avoiding early sexual exposure 4<br>Maintaining personal hygiene 5<br>Avoiding long term oral contraceptive use 6<br>Early treatment of STI 7<br>Regular cervical screening 8**If others specify in KC9 others**<br>Don't know 66	KC9
		Others (specify) …………………………………….	KC9 others
53.	10. Have you ever heard about the vaccine that may prevent cervical cancer?	Yes 1<br>No 0 **If No [2] skip to KS1**	KC10
54.	11. Do you know when the best time to vaccinate against HPV is?<br>At a young age (9-13 years) Reference age from the pilot program in Chitwan and Kaski.	At a young age (9-13 years) 1<br>Before first sexual contact 2<br>After first sexual contact 3<br>After first childbirth 4<br>At any time 5<br>Others 6 **If others specify in KC9 others**<br>Don't know 66	KC11
		Others (specify) …………………………………….	KC11 others

KNOWLEDGE RELATED QUESTIONNAIRE ON SCREENING			
Questions		Responses	Codes
55.	12. Have you ever heard about cervical cancer screening using Visual Inspection with acetic acid (VIA)?	Yes 1<br>No 0 **If No [2] skip to AC1**	KS1
56.	13. If yes, how did you hear or know about VIA?	Television 1<br>Radio 2<br>Newspaper 3<br>Health personnel 4<br>Relatives 5<br>Friends 6<br>FCHV 7<br>Others 8 **If others specify in KS2 others**	KS2
		Others (specify) ……………………………………	KS2 others
57.	14. Do you know when the best time to perform VIA is?	15-49 years old 1<br>60-80 years old 2<br>30-60 years old 3<br>Others 4<br>Don't know 66	KS3
		Others (specify) ……………………………………	KS3 others
58.	15. Do you know how frequently a woman should have VIA performed?	Once 30 years old, every 3-5 years 1<br>Every 3 years 2<br>Every 5 years 3<br>Once in a lifetime, at any age 4<br>When gynecological symptoms appear 5<br>Every 6 months 6<br>Others 7<br>Don't know 66	KS4
		Others (specify) ……………………………………	KS4 others
59.	16. Have you ever heard about any other methods of cervical cancer screening? (Multiple responses)	Pap test 1<br>HPV test 2<br>Others 3 **If others specify in KS5 others**<br>No 4	KS5
		Others (specify) ……………………………………	KS5 others

ATTITUDE							
	**Statement on Attitude towards cervical cancer screening**	**Strongly Disagree**	**Disagree**	**Un decided**	**Agree**	**Strongly agree**	**Code**
60.	Many women are suffering from cervical cancer in Nepal.						AC1
61.	Cervical cancer is a potentially fatal disease.						AC2

62.	Any adult women can acquire cervical cancer.						AC3
63.	Cervical cancer can be detected early through various screening method.						AC4
64.	Screening helps in prevention of cervical cancer.						AC5
65.	Screening causes no harm to the client.						AC6
66.	I will go for screening if it is free of cost.						AC7
67.	HPV vaccination can prevent cervical cancer.						AC8
68.	I will recommend HPV vaccination for my daughters / young girls if it is free of cost.						AC9
69.	I would collect a sample by myself to test for cervical cancer either at a health-care clinic or in home, if I am given instructions on how to collect the sample.						AC10

I. CERVICAL CANCER SCREENING PRACTICE

Questions		Responses	Codes
70.	Has a health-care worker ever tested you for cervical cancer?	Yes 1<br>No 0 **If No [0] skip to SP15**<br>(Do not read) Don't know 66<br>(Do not read) Prefer not to answer 88	SP1
71.	If screened, do you have the screening report?	Yes, showed screening report 1<br>Yes, did not show screening report 2<br>No 0	SP2
72.	If yes, who suggested you to go for cervical cancer screening?	Husband 1 **If Husband [1] skip to SP5**<br>Health worker 2<br>Family member 3<br>Relatives 4<br>Yourself 5<br>Friends 6<br>FCHV 7<br>Mothers group 8<br>**If others specify in SP3 others**	SP3
		Others (specify) ……………………………………	SP3 others
73.	Did your husband support your cervical cancer screening?	Yes 1<br>No 0<br>Did not say anything 3<br>Did not ask husband 4<br>Prefer not to answer 88	SP4

74.	How regularly is your cervical cancer screening (VIA/Pap test/HPV test) done?	Every 2 years 1<br>Every 3 years 2<br>Every 5 years or more 3<br>Not regularly 4<br>Others 5 **If others specify in SP5 others**	SP5
		Others (specify) …………………………………….	SP5 others
75.	At what age, where you first screened for cervical cancer?	Age ☐ ☐<br>(Do not read) Don't know 66<br>(Do not read) Prefer not to answer 88	SP6
76.	When was your last cervical cancer screening done?	Less than 1 year ago 1<br>1-2 years ago 2<br>3-5 years ago 3<br>More than 5 years ago 4<br>Others 5 **If others specify in SP5 others**<br>(Do not read) Don't know<br>(Do not read) Refused 66<br>…………..Year …………. month 88	SP7
		Others (specify) …………………………………….	SP7 others
77.	What is the MAIN reason for you getting your last cervical cancer screening? (check only one)	Part of routine examination 1<br>Follow up on abnormal/ inconclusive result 2<br>Recommended by health-care provider 3<br>Recommended by other source freinds & neighbors 4<br>Experiencing pain or other symptoms 5<br>Others 6<br>Don't know 66<br>Prefer not to answer 88<br>**If others specify in SP5 others**	SP8
		Others (specify) …………………………………….	SP8 others
78.	Where did you receive your last test for cervical cancer?	Private clinic 1<br>Government clinic or hospital 2<br>Community hospital or health institution 3<br>Clinic run by an NGO 4<br>Health camp 5<br>Traditional healer 6<br>Others 7 **If others specify in SP9 others**	SP9
		Others (specify) …………………………………….	SP9 others

79.	What was the result of your last test for cervical cancer?	Did not receive result 1 **If [1] skip to SS1**<br>Normal/negative 2<br>Abnormal/positive 3<br>Suspect cancer 4<br>Inconclusive 5<br>(Do not read) Don't know 66<br>(Do not read) Prefer not to answer 88	SP10
80.	Did you have any follow-up visits because of your last test result?	Yes 1<br>No 2<br>(Do not read) Don't know 66<br>(Do not read) Prefer not to answer 88	SP11
81.	Did you receive any treatment of your cervix because of your last test result?	Yes 1<br>No **2** **If No [2] skip to SP14**<br>(Do not read) Don't know 66<br>(Do not read) Prefer not to answer 88 } **If [66] or [88] skip to SS1**	SP12
82.	Did you receive the treatment of your cervix during the same visit as your last test for cervical cancer?	Yes 1 **If [1] skip to SS1**<br>No 2<br>(Do not read) Don't know 66<br>(Do not read) Prefer not to answer 88 } **If [66] or [88] skip to SS1**	SP13
83.	What is the MAIN reason you did not receive treatment as a result of your last test result? (check only one)<br><br>**Skip to SS1 after this question**	Was not told I needed treatment 1<br>Did not know how/where to get treatment 2<br>Embarrassment 3<br>Too expensive 4<br>Didn't have time 5<br>Clinic too far away 6<br>Poor service quality 7<br>Afraid of the procedure 8<br>Afraid of social stigma 9<br>Cultural beliefs 10<br>Family member would not allow it 11 } **Skip to SS1**<br>Who (specify relationship to respondent)? .....<br>Others 12 **If others specify in SP9 others**<br>(Do not read) Don't know 66<br>(Do not read) Prefer not to answer 88 } **Skip to SS1**	SP14
		Others (specify) …………………………………….	SP14 others
84.	What is the MAIN reason for not receiving cervical cancer screening?	Lack of knowledge about VIA 1<br>Embarrassing 2<br>Economic constraint 3<br>No symptoms/not feeling any discomfort 4<br>Afraid of the result 5	SP15

		Discouraged by husband or other 6<br>Do not like the attitude of medical professionals 7<br>Don't trust the test results 8<br>No access to health facility where the VIA is done 9<br><br>Busy no time 10<br>Hesitation as health worker is a relative 11<br>Screening conducted by a male doctor 12<br>Did not screen due to personal reasons 13<br>Do not feel screening is necessary 14<br>Do not care any consequences from not screening 15<br>Believe in household herbs than screening 16<br><br>**If others specify in SP15 others**	
		Others (specify) ……………………………………	SP15 others
85.	When was the last time you had a gynecological exam done?	Less than one year ago 1<br>More than 1 year ago 2<br>2 years ago 3<br>More than 2 years ago 4<br>3 years ago 5<br>3 to 5 years ago 6<br>More than 5 years ago 7<br>Never undergone gynecological examination umarried 8<br><br>Others **9 If others specify in SP16 others**	SP16
		Others (specify) ……………………………………	SP16 others

SIGN AND SYMPTOMS			
**Questions**		**Responses**	**Codes**
86.	During the last 12 months, did you ever experience pain in your lower abdomen? (except during menstrual period)	Yes 1<br>No 0<br>Prefer not to answer 88<br>**If No [2] skip to SS3**	SS1
87.	If yes, how much did/does this bother you?	Somewhat 1<br>Quite a bit/frequently 2<br>Daily 3	SS2
88.	Do you usually experience pain in the pelvic area?	Yes 1<br>No 0<br>Prefer not to answer 88<br>**If No [2] skip to SS5**	SS3

89.	If yes, how much does this bother you?	Somewhat 1<br>Quite a bit/frequently 2<br>Daily 3	SS4
90.	In the last 12 months, have you experienced foul-smelling genital discharge?	Yes 1<br>No 0<br>Prefer not to answer 88	SS5
91.	In the last 12 months, have you experienced unusual bleeding from your vagina?	Yes 1<br>No 0<br>Prefer not to answer 88 } **If No [2] skip to SSM1**	SS6
92.	Where did you go for checkup when you experienced these symptoms?	Private clinic 1<br>Government clinic or hospital 2<br>Traditional healer 3<br>Clinic run by an NGO 4<br>Others 5<br>**If others specify in SS7 others**	SS7
		Others (specify) ……………………………………	SS7 others

SIGN AND SYMPTOMS (FOR MARRIED WOMEN)			
**Questions**		**Responses**	**Codes**
93.	Do you experience difficulties or pain while having sex?	Yes 1<br>No 0<br>Prefer not to answer 88 } **If No [2] or Prefer not to answer skip to SSM3**	SSM1
94.	If yes, how much does this bother you?	Somewhat 1<br>Quite a bit/frequently 2<br>Daily 3	SSM2
95.	Do you experience bleeding while having sex?	Yes 1<br>No 0<br>Prefer not to answer 88 } **If No [2] or Prefer not to answer skip to MS1**	SSM3
96.	If yes, how much does this bother you?	Somewhat 1<br>Quite a bit/frequently 2<br>Daily 3	SSM4

Sexual activity			
Questions		Response	Code
97.	How old were you when you had sexual intercourse for the very first time?	**years** ☐☐<br>Don't know 66<br>Prefer not to answer 88	MS1
98.	What was this person's relationship to you?	Friend 1<br>Husband 2<br>Boy friend 3<br>Others 4 **If others skip to MS2 others**<br>Prefer not to answer 88	MS2
		Others (specify) ……………………………	MS2 other
99.	When was the last time you had sexual intercourse?	……………….. days ago<br>……………….. weeks ago<br>……………….. months ago<br>……………….. years ago<br>Prefer not to answer 88	MS3
100	What was this person's relationship to you?	Friend 1<br>Husband 2<br>Boy friend 3<br>Others 4 **If others skip to MS4 others**<br>Prefer not to answer 88	MS4
		Others (specify) ……………………………	MS4 others
101	Apart from this person, have you had sexual intercourse with any other person in the last 12 months?	Yes 1<br>No 0	MS5
102	In total, with how many different people have you had sexual intercourse in your lifetime.	None 1<br>One 2<br>Two 3<br>More than two 4<br>Prefer not to answer 88	MS6
